# Myocardial Perfusion Is Impaired and Relates to Cardiac Dysfunction in Patients With Atrial Fibrillation Both Before and After Successful Catheter Ablation

**DOI:** 10.1161/JAHA.118.009218

**Published:** 2018-07-27

**Authors:** Rohan S. Wijesurendra, Alexander Liu, Francesco Notaristefano, Ntobeko A. B. Ntusi, Theodoros D. Karamitsos, Yaver Bashir, Matthew Ginks, Kim Rajappan, Tim R. Betts, Michael Jerosch‐Herold, Vanessa M. Ferreira, Stefan Neubauer, Barbara Casadei

**Affiliations:** ^1^ Division of Cardiovascular Medicine University of Oxford Oxford United Kingdom; ^2^ University of Oxford Centre for Clinical Magnetic Resonance Research Oxford United Kingdom; ^3^ Oxford Heart Centre Oxford University Hospitals NHS Foundation Trust Oxford United Kingdom; ^4^ Brigham and Women's Hospital Harvard Medical School Boston MA

**Keywords:** atrial fibrillation, cardiovascular magnetic resonance imaging, left ventricular function, myocardial blood flow, myocardial perfusion, Atrial Fibrillation, Magnetic Resonance Imaging (MRI)

## Abstract

**Background:**

Atrial fibrillation (AF) is associated with myocardial infarction, and patients with AF and no obstructive coronary artery disease can present with symptoms and evidence of cardiac ischemia. We hypothesized that microvascular coronary dysfunction underlies these observations.

**Methods and Results:**

Myocardial blood flow (MBF) at baseline and during adenosine stress and left ventricular and left atrial function were evaluated by magnetic resonance in 49 patients with AF (25 paroxysmal, 24 persistent) with no history of epicardial coronary artery disease or diabetes mellitus, before and 6 to 9 months after ablation. Findings were compared with those obtained in matched controls in sinus rhythm (n=25). Before ablation, patients with AF had impaired left atrial function and left ventricular ejection fraction and strain indices (all *P*<0.05 versus controls). MBF was impaired in patients both under baseline conditions (1.21±0.24 mL/min per g·[mm Hg·bpm/10^4^]^−1^ versus 1.34±0.28 mL/min per g·[mm Hg·bpm/10^4^]^−1^ in controls, *P*=0.044) and during adenosine stress (2.29±0.48 mL/min per g versus 2.73±0.37 mL/min per g in controls, *P*<0.001). Under baseline conditions, MBF correlated with left ventricular strain and left atrial function (all *P*≤0.001), so that cardiac function was most impaired in patients with the lowest MBF. Baseline and stress MBF remained unchanged postablation (both *P*=ns), and baseline MBF showed similar correlations with functional indices to those present preablation (all *P*≤0.001).

**Conclusions:**

Baseline and stress MBF are significantly impaired in patients with AF but no epicardial coronary artery disease. Reduction in MBF is proportional to severity of left ventricular and left atrial dysfunction, even after successful ablation. Coronary microvascular dysfunction may be a relevant pathophysiological mechanism in patients with a history of AF.

**Clinical Perspective**

**What Is New?**

- Baseline and stress myocardial blood flow, measured by quantitative cardiac magnetic resonance, are significantly impaired in patients with atrial fibrillation but no epicardial coronary artery disease or diabetes mellitus.
- The reduction in baseline myocardial blood flow is proportional to the degree of left ventricular and left atrial functional impairment before catheter ablation.
- Despite successful ablation, myocardial blood flow is unchanged, and the relationship between reduced myocardial blood flow and impaired cardiac function persists.

**What Are the Clinical Implications?**

- Patients with atrial fibrillation appear to have functionally important coronary microvascular dysfunction, which is not reversed following catheter ablation.
- This phenomenon may provide a mechanism to explain why patients with atrial fibrillation can present with chest pain and evidence of cardiac ischemia or necrosis, such as “rate‐related” ST depression or troponin release, in the absence of epicardial coronary artery disease.
- Future studies are needed to determine whether coronary microvascular dysfunction may predict the outcome of ablation and overall prognosis, and hence represent a novel therapeutic target in patients with atrial fibrillation.

## Introduction

Atrial fibrillation (AF) represents a growing worldwide epidemic.^1^ Large‐scale prospective data demonstrate a clear association between AF and incident myocardial infarction in individuals without coronary heart disease, independent of conventional risk factors.^2^ The association is exclusive to non–ST‐elevation myocardial infarction,^3^ implicating an underlying pathophysiology of oxygen supply‐demand imbalance rather than complete thrombotic coronary occlusion. Clinicians are also familiar with patients with AF presenting with chest pain and evidence of cardiac ischemia or necrosis, such as “rate‐related” ST depression or troponin release, in the absence of significant epicardial coronary artery disease.^4^, ^5^ Despite these intriguing observations, few studies have systematically investigated myocardial perfusion in patients with AF.^6^, ^7^

Cardiac magnetic resonance (CMR) is a validated, noninvasive, and accurate method for detecting myocardial hypoperfusion^8^ and can also quantify myocardial blood flow (MBF).^9^ We hypothesized that CMR would demonstrate microcirculatory dysfunction and impaired myocardial perfusion in patients with paroxysmal or persistent AF who have no significant epicardial coronary artery disease or diabetes mellitus and that these findings would relate to both left atrial (LA) dysfunction and the reduction in left ventricular (LV) function that is present even when the ventricular rate is well controlled.^10^, ^11^ We used adenosine stress to evaluate myocardial perfusion reserve and quantitatively determine baseline and stress MBF, both before and after catheter ablation, to assess the effect of restoration of sinus rhythm (SR).

## Methods

The study complied with the Declaration of Helsinki; the research protocol was approved by the local Research Ethics Committee, and all subjects gave written informed consent. R.S.W. had full access to all the data in the study and takes responsibility for its integrity and the data analysis. The data, analytic methods, and study materials will be made available to other researchers for purposes of reproducing the results or replicating the procedure on written request to the corresponding author.

### Study Population

Patients undergoing first‐time catheter ablation of symptomatic paroxysmal or persistent AF were screened for eligibility. Individuals with epicardial coronary artery disease, previous coronary intervention or cardiac surgery, uncontrolled ventricular rate (>90 bpm) or arterial blood pressure (BP) (>160/90 mm Hg), significant valvular disease, amyloidosis, uncontrolled thyroid disease, systemic inflammatory disease, diabetes mellitus, or obstructive sleep apnea were not enrolled. Further exclusion criteria were contraindications to CMR or to administration of gadolinium and/or adenosine.

Control subjects in SR were recruited via poster advertising. Exclusion criteria included those for patients and a history of palpitations or arrhythmia. Controls were selected to match patients for age, sex, body mass index, and arterial BP on an overall group basis.

Patients were scanned ≤4 weeks before ablation and 6 to 9 months after ablation. Patients in SR 3 months postablation underwent routine twice‐daily and additional symptom‐guided ECG event monitoring (OMRON HeartScan HCG‐801, Omron Healthcare, Bannockburn, IL) for a further 3 months, and 7‐day Holter monitoring following the postablation CMR, to detect asymptomatic AF recurrence. Controls underwent a single CMR scan. All subjects avoided potential adenosine antagonizers (eg, caffeine) for ≥24 hours before scans. Clinical management (including index ablation strategy, medical therapy, and decisions regarding repeat ablation or cardioversion) was at the discretion of the responsible physician. Pulmonary vein isolation was the mainstay of the index ablation procedure and was undertaken in all patients.

### CMR Imaging

CMR was performed at 1.5 Tesla (Siemens Avanto, Siemens Healthcare, Erlangen, Germany). CMR imaging was undertaken using a 32‐channel phased‐array coil with the subject supine. Images were acquired during end‐expiration to minimize the effects of respiratory motion. The established techniques of cine and adenosine stress/rest perfusion were applied as previously described^10^, ^12^ and are detailed below. Strain indices were assessed by the LV tissue‐tracking method.^13^ Positive strain values describe myocardial thickening, whereas negative values describe myocardial shortening.

All data sets were anonymized and placed in a random order for contouring. Contours were placed by operators not involved with data acquisition and blinded to clinical characteristics, intrascan rhythm, and study time point. Splenic switch‐off was assessed visually—this technique has recently been described as a method of identifying stress adequacy in adenosine perfusion CMR.^14^ Rate pressure product (RPP) was calculated separately at baseline and peak stress, by multiplying HR by systolic BP.

### Cine Imaging

Cines were acquired using steady‐state free precession imaging. Scan parameters were typically voxel size 2.0×2.0×8.0 mm, field of view=380×380 mm, repetition time/echo time (TR/TE) 39.6/1.12 milliseconds, flip angle 55°, matrix 192×192, Generalized Autocalibrating Partial Parallel Acquisition (GRAPPA)=3, 24 reference lines, segments=15, concatenations=1. Pilot images were initially acquired and used to plan and acquire horizontal long axis, vertical long axis, and left ventricular outflow tract long‐axis and short‐axis stack images.

Cines were acquired with retrospective gating for patients in SR at the time of the scan and with arrhythmia sorting as a first‐choice method for patients in AF at the time of the scan. For the minority of patients in AF in whom acceptable images could not be obtained, prospectively triggered cines were acquired instead.

Cine image analysis was conducted offline with cmr42 postprocessing software (version 5.1.1, Circle Cardiovascular Imaging Inc, Calgary, ON, Canada). Left atrial maximal volume (LA_max_) and minimal volume (LA_min_) were determined with the biplane area‐length method and used to calculate total LA emptying fraction=(LA_max_−LA_min_)/LA_max_. Strain indices reported are global myocardial values determined from 2‐dimensional analysis of the entire stack of short‐axis cine images.

### Perfusion Imaging

First‐pass perfusion imaging was performed during peak adenosine stress (140 μg/kg per min, intravenously for ≥3 minutes) with an intravenous bolus of gadolinium (0.03 mmol/kg; Dotarem, Guerbet, Villepinte, France), followed by a 15‐mL saline flush. Rest perfusion images were acquired with an identical gadolinium bolus ≥15 minutes after adenosine discontinuation. Under both conditions, images were acquired on every cardiac cycle using a T1‐weighted fast (spoiled) gradient echo sequence. Scan parameters were typically voxel size 2.8×2.3×10.0 mm, field of view=360×270 mm, matrix 96×160, TR/TE =160.76/1.05 milliseconds, flip angle 12°, inversion time 100 milliseconds, GRAPPA=2, 18 reference lines, measurements=50, segments=57, concatenations=1, phases=1, 1 shot per slice, bandwidth 651 Hz/Px.

### Absolute Myocardial Perfusion Quantification

Quantitative perfusion analysis was performed as previously described^9^; briefly, MBF was determined independently at baseline and under adenosine stress by model‐independent deconvolution of signal intensity curves with an arterial input function measured in the LV blood pool, with explicit accounting for any delay in the arrival of the tracer. Fitting quality of MBF was assessed in a blinded fashion for each myocardial segment; MBF values derived from segments with good fitting were averaged to derive a global per‐subject MBF value, which was used for all analyses.

Baseline MBF is closely related to baseline RPP^15^; thus, baseline MBF was corrected for baseline RPP using the formula baseline MBF_corrected_=baseline MBF/(baseline RPP/10 000), to account for differences in cardiac work, as previously described.^6^ In accordance with previous studies, adenosine‐stress–induced MBF was not corrected because this parameter is grossly independent of RPP.^16^ The myocardial perfusion reserve index was calculated by dividing the numerical value of stress MBF by the numerical value of baseline MBF_corrected_.

### Statistical Analysis

Power calculations were based on prior data reporting a baseline MBF of 0.95±0.19 mL/(min·mL) in AF patients and 1.14±0.22 mL/(min·mL) in matched controls, and a MBF during adenosine stress of 2.07±0.80 mL/(min·mL) in AF patients and 3.33±0.78 mL/(min·mL) in matched controls.^6^ We calculated that inclusion of at least 48 patients and 24 controls (2:1 allocation ratio) would give >90% power to detect a difference of ≥15% in baseline MBF and ≥20% in stress MBF, respectively, between patients and controls (2‐sided α=0.05). Paired analysis of 30 patients before and after ablation gave >90% power to detect a change of ≥15% in baseline MBF and ≥25% in stress MBF, respectively (2‐sided α=0.05, between group correlation=0.5).

Analyses were performed using IBM SPSS Statistics for Windows version 24.0.0.1 (IBM Corp, Armonk, NY) and GraphPad Prism version 6 (GraphPad Software, San Diego, CA). Data distribution was assessed using the Kolmogorov‐Smirnov test. Normally distributed data were compared using the Student t tests (paired when appropriate) or 1‐way ANOVA. Non–normally distributed unpaired data were compared using the Mann‐Whitney U test or the Kruskal‐Wallis test. Non–normally distributed paired data were compared using the Wilcoxon signed‐rank test. The chi‐squared test was used to compare proportions, and the Pearson r or Spearman ρ to report linear correlations between normally distributed or non–normally distributed parameters, respectively. All tests were 2‐tailed, and *P*<0.05 (after Bonferroni adjustment for multiple comparisons, when appropriate) were considered significant. Data are shown as mean±SD or median and interquartile range (IQR).

## Results

A total of 74 subjects were included: 49 patients with AF (25 paroxysmal, 24 persistent), and 25 controls in SR. Patients and controls were well matched for all characteristics except treatment (Table 1). Patients had a median CHA_2_DS_2_‐VASc score of 1 (IQR 0‐2), and median time from first AF diagnosis was 3.9 years (IQR 2.0‐7.4 years).

**Table 1:** Baseline Characteristics of the Study Groups

	Patients With AF (n=49)	Controls in SR (n=25)	*P* Value
Age, y	67 (57‐70)	64 (63‐66)	0.60
Male, n (%)	37 (76)	19 (76)	0.96
BMI, kg/m^2^	28±5	27±3	0.36
Pulse, bpm	60 (54‐78)	66 (60‐68)	0.98
SBP, mm Hg	130 (124‐146)	137 (124‐155)	0.36
DBP, mm Hg	81±12	80±11	0.86
ACEI/ARB, n (%)	22 (45)	1 (4)	···
β‐blocker/Sotalol, n (%)	33 (67)	0 (0)	···
Calcium‐channel blocker, n (%)	12 (24)	4 (16)	···
Flecainide, n (%)	12 (24)	0 (0)	···
Digoxin, n (%)	7 (14)	0 (0)	···
Amiodarone, n (%)	4 (8)	0 (0)	···

Values are presented as mean±SD, median (IQR), or number (percentage). ACEI indicates angiotensin converting enzyme inhibitor; AF, atrial fibrillation; ARB, angiotensin II receptor blocker; BMI, body mass index; DBP, diastolic blood pressure; IQR, interquartile range; SBP, systolic blood pressure; SR, sinus rhythm.

### Preablation LV and LA Indices and Perfusion Data

Compared with controls, patients had significantly larger LA (*P*<0.001) and impaired LV systolic function, as measured by ejection fraction, peak systolic circumferential strain, and peak systolic radial strain (all *P*<0.001), LV diastolic function by peak diastolic radial strain rate (*P*<0.05), and LA emptying fraction (*P*<0.001; Table 2). Consistent with exclusion of individuals with uncontrolled BP, LV mass index was within the CMR normal range^17^ and similar between patients and controls. There were no visual perfusion defects in any study subject on stress or baseline perfusion imaging, consistent with the exclusion of patients with known epicardial coronary artery disease.

**Table 2:** LV and LA Indices in the Study Groups

	Patients With AF (n=49)	Controls in SR (n=25)	*P* Value
LV peak systolic radial strain, %	31±9	40±6	<0.001^a^
LV peak systolic circumferential strain, %	−16±3	−19±2	<0.001^a^
LV peak diastolic radial strain rate, s^−1^	−2.0±0.6	−2.2±0.4	0.039^a^
LV ejection fraction, %	63 (53–66)	69 (65–72)	<0.001^a^
LV mass index, g/m^2^	61±12	56±11	0.09
LA maximal volume, mL	86 (79–124)	76 (65–83)	0.001^a^
LA emptying fraction, %	31 (18–49)	60 (52–63)	<0.001^a^

Values are presented as mean±SD or median (IQR). AF indicates atrial fibrillation; IQR, interquartile range; LA, left atrial; LV, left ventricular; SR, sinus rhythm.

a *P* <0.05.

Absolute MBF quantification could be undertaken successfully at the preablation time point in 42 patients with AF (23 paroxysmal and 19 persistent) and 25 controls in SR. Rejection of all myocardial segments due to poor fitting quality occurred in 6 patients in AF and in 1 patient in SR during CMR. In the remaining subjects a total of 549 segments were rejected out of the total available 2412 segments (23% rejection rate); the segmental rejection rate was 18% in scans undertaken in SR and 34% in scans undertaken in AF (*P*<0.001).

**Figure 1:**
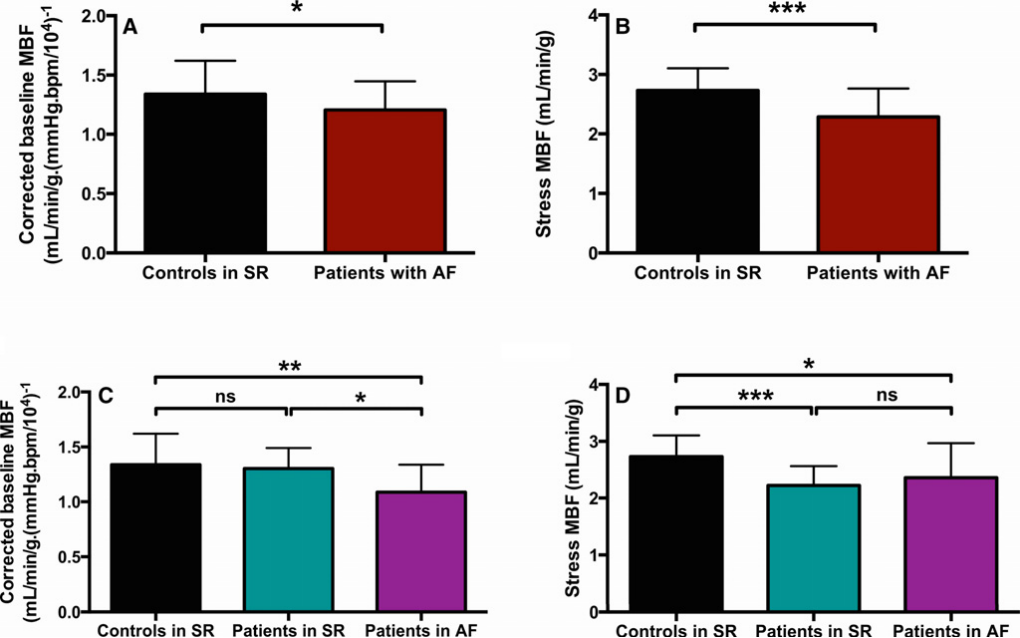
MBF indices preablation. Patients with atrial fibrillation (AF; n=42) have significantly lower baseline myocardial blood flow (MBF; A) and adenosine‐stress–induced MBF (B) preablation compared with matched control subjects in sinus rhythm (SR; n=25). The reduction in baseline MBF is driven mainly by patients with a rhythm of AF at the time of imaging (C), whereas stress MBF is impaired in patients with AF compared with controls irrespective of the rhythm at the time of imaging (D). Numbers in each group in panels C and D are as follows: controls in SR, n=25; patients in SR, n=23; patients in AF, n=19. Data are presented as mean±SD; ns indicates nonsignificant; *P<0.05, **P<0.01, ***P<0.001.

Baseline and stress hemodynamic and MBF data in patients and controls are summarized in Table 3 and Figure 1 A, B. As expected, absolute baseline MBF was correlated with baseline RPP in all subjects (*r*=0.622, *P*<0.001), with a similar correlation in controls and patients (*r*=0.647 in controls in SR and *r*=0.602 in AF patients, both *P*<0.001). Compared with controls, AF patients had significantly lower baseline and stress MBF. At the time of the CMR scan, 23 AF patients awaiting ablation were in SR, whereas 19 were in AF.

**Table 3:** Hemodynamic and MBF Indices in Preablation Patients and Controls

	1	2	3	*P* Value (1 vs 2)	*P* Value (1 vs 3)	*P* Value (2 vs 3)
	Patients in SR During CMR (n=23)	Patients in AF During CMR (n=19)	Controls in SR (n=25)			
Baseline HR, bpm	54±8	68±16	64±11	0.001^a^	0.025^a^	0.693
Baseline systolic BP, mm Hg	137±16	141±20	142±18	1.000	1.000	1.000
Baseline RPP, bpm·mm Hg	7400±1300	96±2600	9100±2400	0.005^a^	0.025^a^	1.000
Stress HR, bpm	74 (65‐79)	72 (60‐77)	96 (84‐101)	1.000	0.004^a^	0.001^a^
Stress systolic BP, mm Hg	132±17	139±22	138±16	0.682	0.941	1.000
Stress RPP, bpm·mm Hg	10 000±2900	9800±2200	12 800±2600	1.000	0.003^a^	0.003^a^
ΔRPP, bpm·mm Hg	2500±2100	200±1300	3300±1900	<0.001^a^	0.543	<0.001^a^
Uncorrected baseline MBF, mL/(min·g)	0.96±0.17	1.01±0.27	1.19±0.27	1.000	0.005^a^	0.055
Baseline MBF, mL/(min·g)·(mm Hg·bpm/10^4^)^−1^	1.30±0.19	1.09±0.25	1.34±0.28	0.016^a^	1.000	0.003^a^
Stress MBF, mL/(min·g)	2.22±0.34	2.36±0.60	2.73±0.37	0.919	0.001^a^	0.024^a^
Quantitative MPR, index	1.74±0.38	2.28±0.71	2.11±0.44	0.004^a^	0.047^a^	0.844

Values are presented as mean±SD or median (IQR). AF indicates atrial fibrillation; BP, blood pressure; CMR, cardiac magnetic resonance; HR, heart rate; IQR, interquartile range; MBF, myocardial blood flow; MPR, myocardial perfusion reserve; RPP, rate‐pressure product; SR, sinus rhythm.

a *P* <0.05.

Lower baseline MBF values were observed in patients in AF during the CMR scan (Figure 1C); as expected, the majority (84%) of this group had a history of persistent AF (rather than paroxysmal AF; Table 4). By contrast, stress MBF was impaired in all patients, irrespective of the intrascan rhythm or the history of paroxysmal or persistent AF (Figure 1D). Myocardial perfusion reserve was reduced in patients in SR during the CMR scan but was “pseudonormal” in patients in AF during the CMR scan due to relatively matched reductions in both baseline and stress MBF compared with controls (Table 3).

**Table 4:** Baseline Characteristics of Patients Classified by Rhythm During the Preablation CMR Scan

	Patients in SR (n=24)	Patients in AF (n=25)	*P* Value
Paroxysmal AF, n (%)	21 (88%)	4 (16%)	<0.001^a^
Persistent AF, n (%)	3 (13%)	21 (84%)	<0.001^a^
Age, y	67 (58–70)	64 (56–70)	0.76
Male, n (%)	18 (75)	19 (76)	0.94
BMI, kg/m^2^	27±3	29±6	0.21
Pulse, bpm	55 (48–60)	72 (66–90)	<0.001^a^
SBP, mm Hg	129 (124–146)	135 (126–146)	0.62
DBP, mm Hg	77±11	85±11	0.017^a^
ACEI/ARB, n (%)	6 (25)	16 (64)	0.006^a^
β‐blocker/Sotalol, n (%)	15 (63)	18 (72)	0.48
Calcium‐channel blocker, n (%)	6 (25)	6 (24)	0.94
Flecainide, n (%)	11 (46)	1 (4)	0.001^a^
Digoxin, n (%)	0 (0)	7 (28)	0.005^a^
Amiodarone, n (%)	2 (8)	2 (8)	0.97

ACEI indicates angiotensin‐converting enzyme inhibitor; AF, atrial fibrillation; ARB, angiotensin II receptor blocker; BMI, body mass index; CMR, cardiac magnetic resonance; DBP, diastolic blood pressure; SBP, systolic blood pressure; SR, sinus rhythm.

a *P* <0.05.

Positive splenic switch‐off (ie, evidence of adequate stress) was present in 88% of scans in patients and 92% of scans in controls (*P*=0.58). All subjects experienced at least 1 adenosine‐related symptom (eg, chest tightness, dyspnea, flushing). The impairment in baseline and stress MBF in patients compared with controls remained significant when subjects without splenic switch‐off were excluded from analysis (Table 5). There were no significant differences in either baseline or stress MBF between patients receiving and those not receiving β‐blocker or angiotensin‐converting enzyme inhibitor/angiotensin II receptor blocker medications (*P*=ns for all comparisons).

**Table 5:** Preablation MBF in Patients and Controls Excluding Subjects Without Splenic Switch‐Off

	Patients Preablation (n=36)	Controls in SR (n=23)	*P* Value
Baseline MBF, mL/(min·g)·(mm Hg·bpm/10^4^)^−1^	1.21±0.26	1.38±0.26	0.016^a^
Stress MBF, mL/(min·g)	2.33±0.37	2.74±0.36	<0.001^a^

MBF indicates myocardial blood flow; SR, sinus rhythm.

a *P* <0.05.

### Relationship Between MBF and Cardiac Function

**Figure 2:**
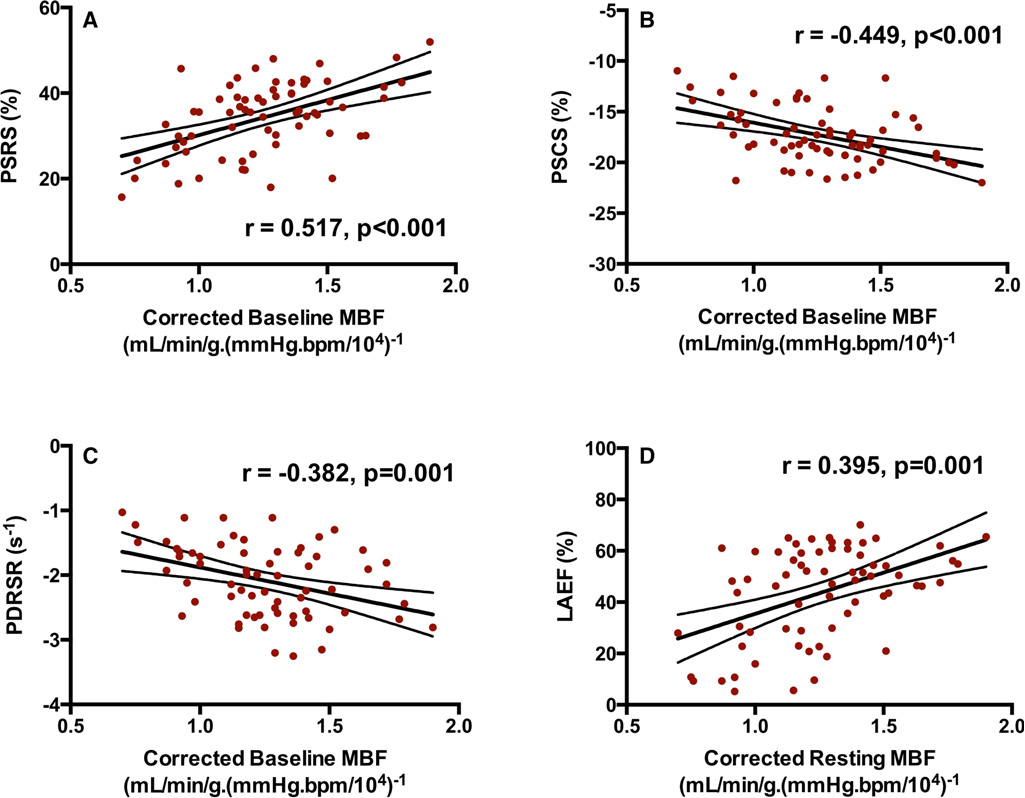
Relationships between baseline myocardial blood flow (MBF) and indices of cardiac function. Relationships between baseline MBF and left ventricular (LV) peak systolic radial strain (PSRS; A), LV peak systolic circumferential strain (PSCS; B), LV peak diastolic radial strain rate (PDRSR; C), and left atrial (LA) emptying fraction (LAEF; D) in patients with atrial fibrillation preablation (n=42) and control subjects in sinus rhythm (n=25). Pearson r or Spearman rho, *P* value, line of best fit, and 95% confidence bands are also shown in each case.

Baseline MBF was positively correlated with baseline LV peak systolic radial strain (Figure 2A) and inversely correlated with LV peak systolic circumferential strain and LV peak diastolic radial strain rate (Figure 2B and 2C, respectively). Similarly, baseline MBF was positively correlated with baseline LA emptying fraction (Figure 2D). The direction of correlation supported an association between higher MBF and better LV and LA function in each case (all *P*≤0.001). Addition of clinical variables (including age, sex, body mass index, systolic BP, and diastolic BP) to linear regression models did not materially alter the relationship between baseline MBF and each of these markers of cardiac function (data not shown).

### Effect of Ablation

Ablation was undertaken in 47 patients; the scheduled ablation was canceled for clinical reasons in 2 patients. Radiofrequency ablation was used in 31 patients (66%), cryoballoon ablation in 14 patients (30%), and laser balloon ablation in 2 patients (4%). In the time span between the end of the 3‐month blanking period and the 7‐month visit, 8 patients (17%) underwent an attempt at electric cardioversion, and 3 patients (6%) underwent a second ablation procedure as a result of recurrence of AF or left atrial tachycardia. Following ablation, the Holter‐determined AF burden fell significantly (median [IQR] from 46% [1% to 100%] to 0% [0% to 0%], *P*<0.001), and 20 of 43 patients (47%) who underwent postablation CMR had no episodes of symptomatic or asymptomatic recurrent AF on either Holter or intermittent ECG monitoring. The proportion of patients free of recurrent AF after ablation was similar between those individuals with paroxysmal AF and persistent AF (43% versus 50%, *P*=0.67).

**Figure 3:**
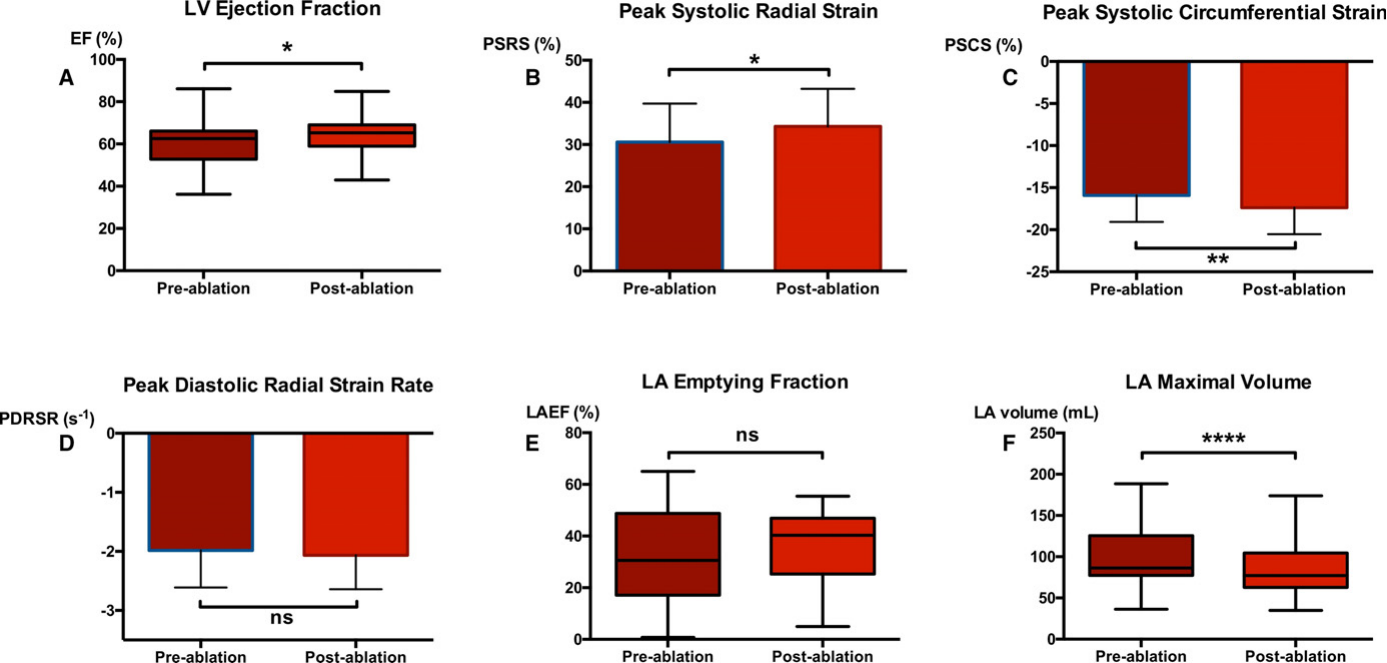
Pre‐ and postablation indices of left ventricular (LV) systolic and diastolic function and left atrial (LA) volume and function. Comparison of paired (n=43) pre‐ and postablation LV ejection fractions (EF; A), LV peak systolic radial strains (PSRS; B), LV peak systolic circumferential strains (PSCS; C), LV peak diastolic radial strain rates (PDRSR; D), LA emptying fractions (LAEF; E), and LA maximal volumes (F). Data are presented as mean±SD or median/IQR/range; *P<0.05, ****P<0.0001. IQR indicates interquartile range; ns, nonsignificant.

Ablation was associated with a significant improvement in baseline LV ejection fraction, LV peak systolic radial strain, and LV peak systolic circumferential strain (Figure 3A through 3C); however, all of these parameters remained impaired when relative to control subjects in SR (all *P*<0.05). LV peak diastolic radial strain rate was unchanged after ablation (Figure 3D), as was LA emptying fraction (Figure 3E), despite a significant reduction in LA maximal volume (Figure 3F).

**Figure 4:**
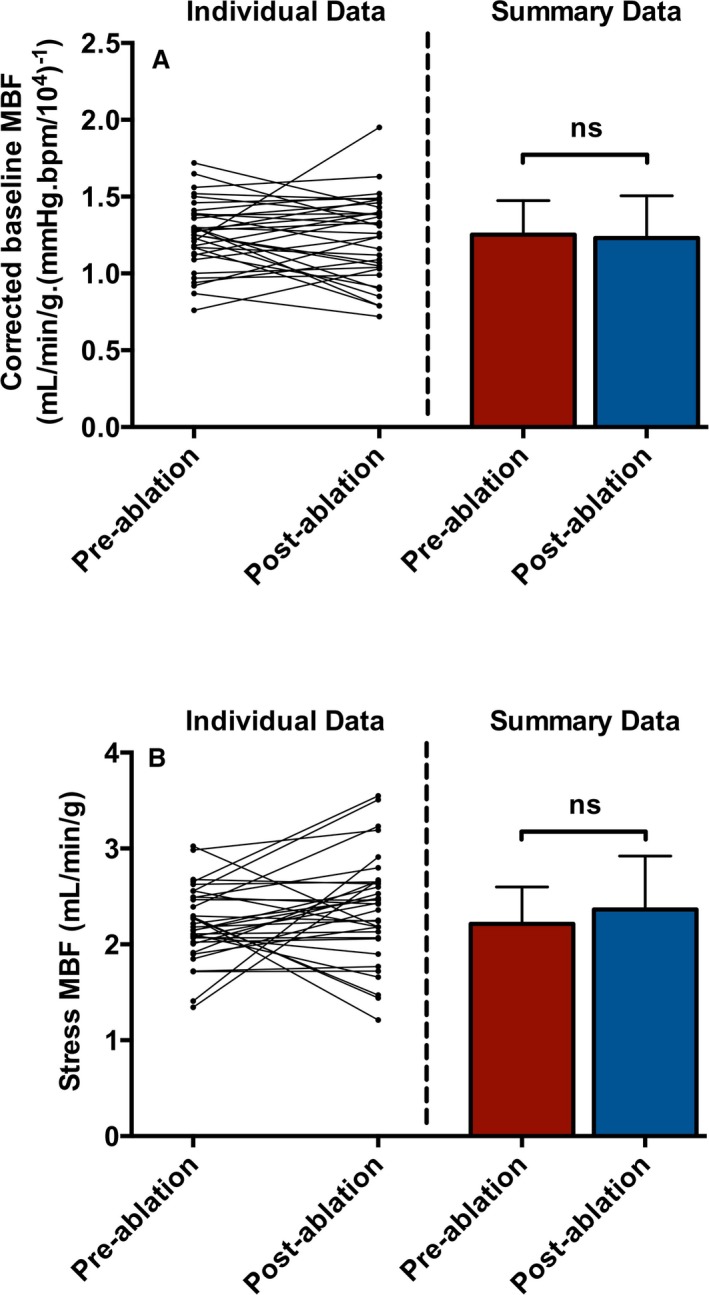
Paired pre‐ and postablation MBF. Individual (left panels) and summary (right panels) results from paired pre‐ and postablation analyses of baseline MBF (A) and stress MBF (B) in patients with atrial fibrillation (n=33). Neither parameter changes significantly (*P*=0.59 for baseline MBF, and *P*=0.16 for stress MBF). MBF indicates myocardial blood flow.

Paired pre‐ and postablation MBF results were available in 33 patients. Within this group, 29 patients (88%) were in SR at the postablation CMR scan (compared with 22 patients or 67% at the preablation CMR scan), AF burden fell significantly (median [IQR] from 11% [0% to 100%] to 0% [0% to 0.1%], *P*=0.001), and 15 of 33 patients (45%) had no episodes of recurrent AF. Baseline and stress MBF after ablation were unchanged compared with preablation values in paired analyses (Figure 4). This finding remained consistent when patients with ≥1 episode of recurrent AF postablation were excluded (Table 6) and when patients in AF at the preablation CMR were also excluded (Table 7). Across all postablation patients in whom MBF could be determined (n=40), stress MBF remained significantly impaired compared with controls; by contrast, baseline MBF was similar between these groups, consistent with the majority of patients (88%) being in SR during the postablation CMR scans (Table 8).

**Table 6:** Paired Pre‐ and Postablation MBF in Patients With No Recurrent AF Postablation

	Preablation (n=15)	Postablation (n=15)	*P* Value
Baseline MBF, mL/(min·g)·(mm Hg·bpm/10^4^)^−1^	1.24±0.24	1.22±0.23	0.72
Stress MBF, mL/(min·g)	2.05±0.33	2.25±0.53	0.28

MBF indicates myocardial blood flow.

**Table 7:** Paired Pre‐ and Postablation MBF in Patients in SR at the Preablation CMR and With No Recurrent AF Post‐Ablation

	Preablation (n=9)	Postablation (n=9)	*P* Value
Baseline MBF, mL/(min·g)·(mm Hg·bpm/10^4^)^−1^	1.29±0.17	1.33±0.16	0.32
Stress MBF, mL/(min·g)	2.07±0.36	2.32±0.50	0.24

AF indicates atrial fibrillation; CMR, cardiac magnetic resonance; MBF, myocardial blood flow; SR, sinus rhythm.

**Table 8:** Hemodynamic and MBF Indices in Postablation Patients and Controls

	Patients Postablation (n=40)	Controls in SR (n=25)	*P* Value
Baseline RPP, bpm·mm Hg	8200±1400	9100±2400	0.09
Stress RPP, bpm·mm Hg	9800±2600	12 700±2500	<0.001^a^
ΔRPP, bpm·mm Hg	1600±2400	3300±1900	0.007^a^
Uncorrected baseline MBF, mL/min per g	1.01±0.25	1.19±0.27	0.009^a^
Baseline MBF, mL/(min·g)·(mm Hg·bpm/10^4^)^−1^	1.25±0.29	1.34±0.28	0.21
Stress MBF, mL/(min·g)	2.37±0.58	2.73±0.37	0.008^a^
Quantitative MPR, index	1.97±0.53	2.11±0.44	0.27

Stress MBF and Quantitative MPR data available on 39 of 40 patients. MBF indicates myocardial blood flow; MPR, myocardial perfusion reserve; RPP, rate‐pressure product; SR, sinus rhythm.

a *P* <0.05.

**Figure 5:**
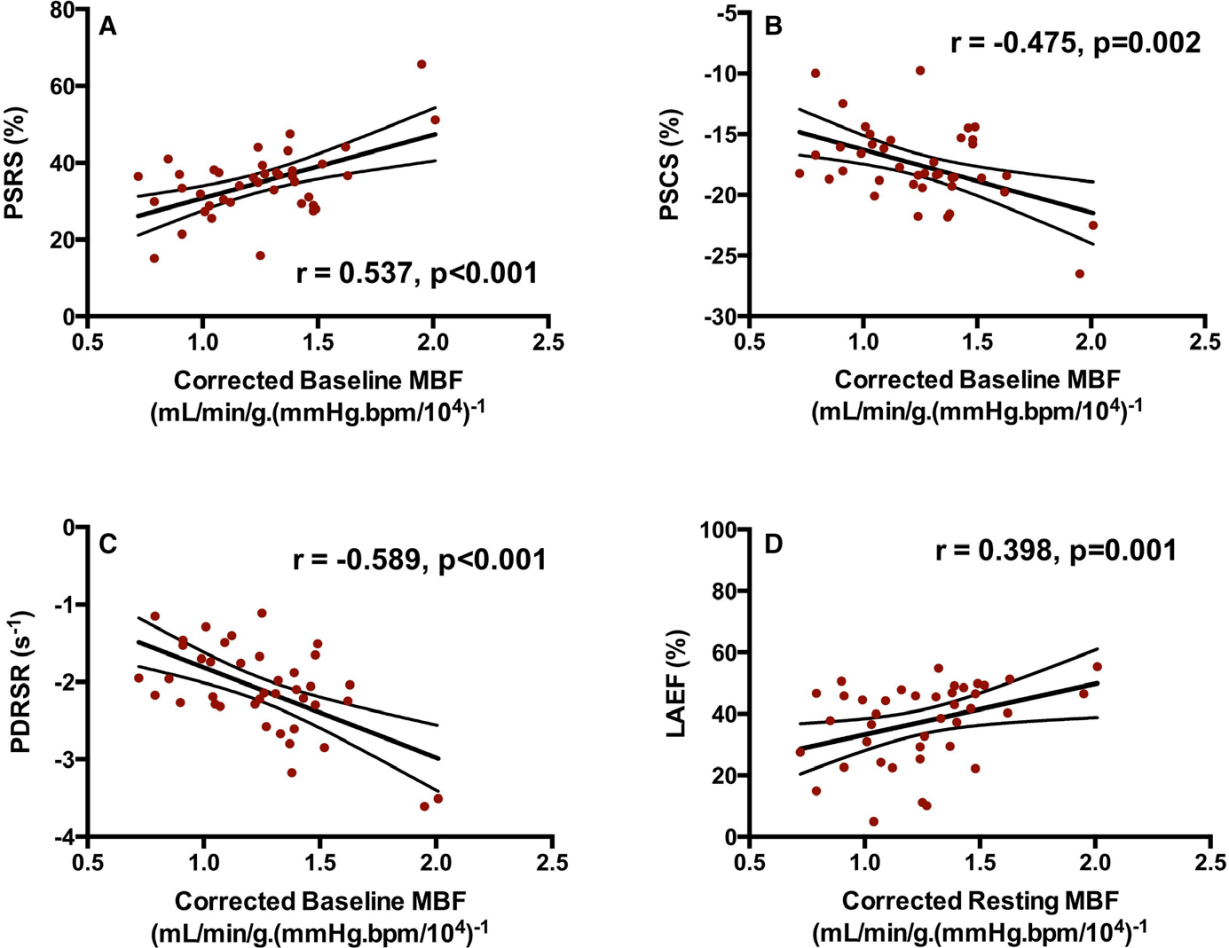
Relationships between baseline myocardial blood flow (MBF) and indices of cardiac function after ablation. Relationships between baseline MBF and left ventricular (LV) peak systolic radial strain (PSRS; A), LV peak systolic circumferential strain (PSCS; B), LV peak diastolic radial strain rate (PDRSR; C), and left atrial (LA) emptying fraction (LAEF; D) after ablation (n=40 patients with baseline MBF data postablation). Pearson r or Spearman ρ, *P* value, line of best fit, and 95% confidence bands are also shown in each case.

**Figure 6:**
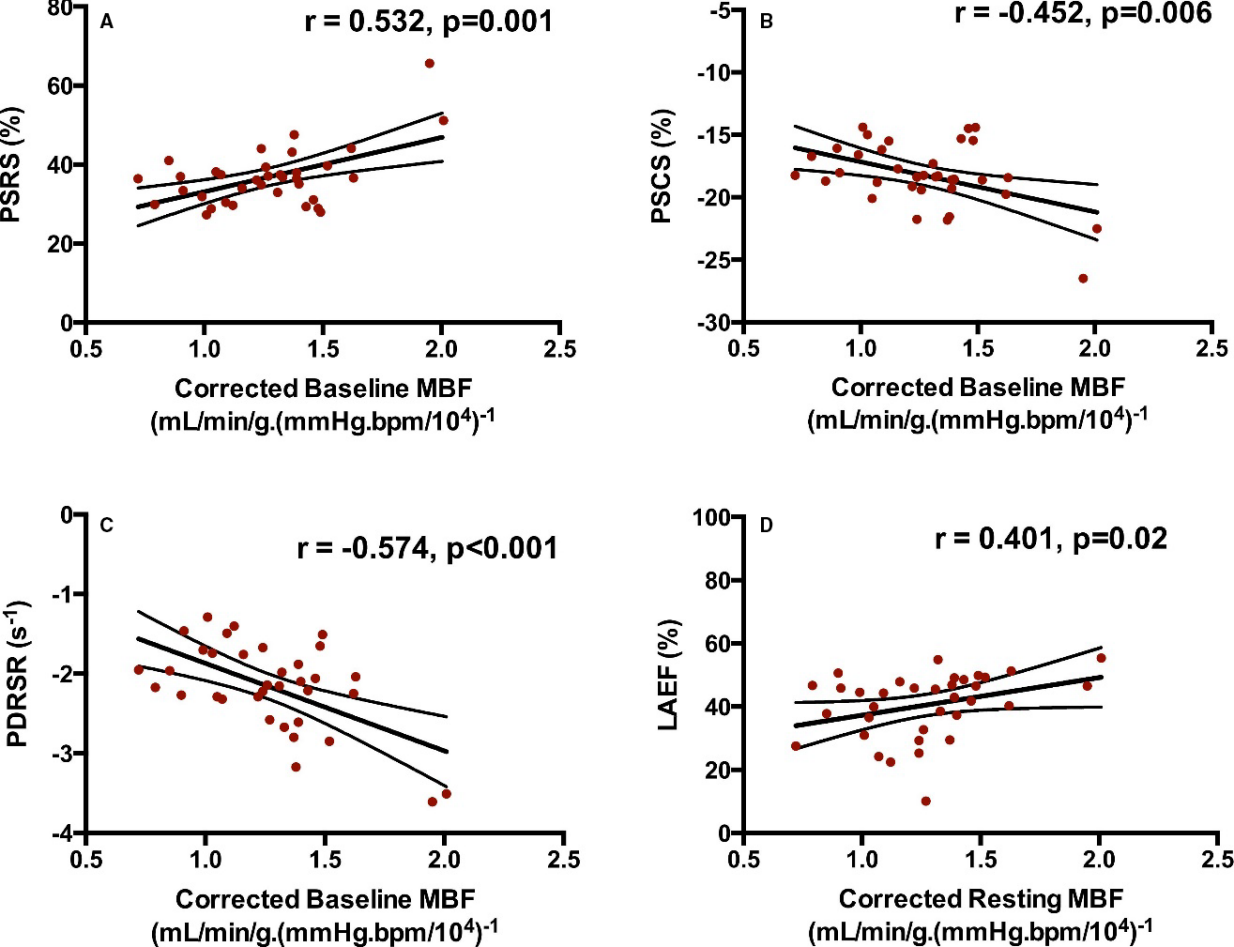
Relationships between baseline myocardial blood flow (MBF) and indices of cardiac function in patients in sinus rhythm during the postablation cardiac magnetic resonance scan. Relationships between corrected baseline myocardial blood flow and left ventricular (LV) peak systolic radial strain (PSRS; A), LV peak systolic circumferential strain (PSCS; B), LV peak diastolic radial strain rate (PDRSR; C), and left atrial emptying fraction (LAEF; D) in patients in sinus rhythm during the postablation cardiac magnetic resonance scan (n=35). Pearson r or Spearman ρ and the respective *P* values for the associations are shown in each case. Lines of best fit and 95% confidence bands are also shown.

As observed before ablation, postablation baseline MBF remained positively correlated with LV peak systolic radial strain and LA emptying fraction and inversely correlated with LV peak systolic circumferential strain and LV peak diastolic radial strain rate (all *P*≤0.001; Figure 5). These correlations remained significant when analysis was restricted to patients in SR at postablation CMR (n=35, all *P*<0.05; Figure 6).

## Discussion

This prospective study used advanced CMR techniques to quantify myocardial perfusion at baseline and during adenosine stress in patients with a diagnosis of AF and no significant epicardial coronary disease, and in matched controls in SR. In addition to showing reduced baseline and stress MBF in patients with AF compared with controls, these data indicate for the first time that reduced baseline MBF is proportional to the degree of LV and LA functional impairment. Despite successful ablation MBF was unchanged, and the relationship between MBF and cardiac function persisted. These findings imply that AF is associated with functionally important coronary microvascular dysfunction and that restoration of SR by ablation alone is insufficient to reverse this process.

### MBF Is Impaired in Patients With AF and Relates to LV and LA Dysfunction

Our study adds new and unique insight regarding the functional relevance of abnormalities in MBF in patients with AF. Interestingly, only patients in AF during the preablation CMR (a high percentage of whom had a diagnosis of persistent AF) had reduced baseline MBF relative to controls in SR or to patients with a diagnosis of (mostly paroxysmal) AF who were in SR at the time of the scan. These findings are in keeping with experimental data demonstrating that the increase in MBF that accompanies the induction of AF is insufficient to compensate for the increase in cardiac workload.^7^ Moreover, the presence of persistent AF may indicate a more advanced underlying cardiomyopathy characterized by significant microvascular and myocardial dysfunction even in the absence of stress, leading to (and being exacerbated by) the sustained arrhythmia.

We found highly significant and moderately strong correlations between baseline MBF and both LV strain parameters and LA emptying fraction, which persisted after ablation. These findings suggest that microvascular dysfunction could affect both LV and LA perfusion^18^, ^19^ and could directly contribute to the substrate that initiates and maintains AF.

Our study supports previous findings of impaired stress MBF in male patients with persistent AF determined using positron emission tomography^6^ and extends the investigation to female patients and those with paroxysmal AF who were in SR at the time of imaging. Stress MBF was lower in all AF patients, irrespective of the rhythm during the preablation CMR. This difference also persisted after ablation, even in the subgroup of patients with no evidence of recurrent AF on prolonged ambulatory monitoring, suggesting that the previously reported improvement in this parameter in a very small number of patients following cardioversion^6^ may have been due to the play of chance. These novel findings imply that the reduction in stress MBF in patients with paroxysmal or persistent AF is not a direct effect of the arrhythmia itself but may reflect an underlying coronary endothelial dysfunction.^11^

As reported previously,^6^ we did not find significant differences in myocardial perfusion reserve between patients with AF and matched control subjects in SR. This is not surprising as in patients with hypertrophic or dilated cardiomyopathy (and no epicardial coronary artery disease),^20^, ^21^ stress MBF has been shown to be superior to perfusion reserve for uncovering coronary microvascular dysfunction and predicting adverse cardiac events.^22^

### Clinical Implications and Future Directions

We previously demonstrated impaired LV energetics that persists despite successful ablation, suggesting that apparently “lone” AF may actually be the consequence of an occult cardiomyopathy.^10^ It is possible that coronary microvascular dysfunction is part of, or even responsible for, the same underlying disease process. Future studies are needed to determine whether these parameters predict the outcome of ablation and whether global risk factor management (beyond ablation alone) may improve both myocardial energetics and perfusion in patients with AF. Such a link would provide further evidence for the mechanistic importance of coronary microvascular and energetic dysfunction in AF, and confirm their validity as therapeutic targets.

The observed reduction in stress MBF would be expected to be important in determining exercise capacity and other parameters of functional cardiac reserve. Interestingly, reduced exercise capacity and functional status are closely related to the risk of developing AF and precede the onset of the arrhythmia.^23^ The hypothesis that impaired stress MBF underlies the reduction in exercise capacity in individuals at risk of AF and predicts onset of the arrhythmia merits further investigation.

### Limitations

As expected, vasoactive and antiarrhythmic medication use was more common in patients with AF than in control subjects. Medications were not withdrawn before scans to avoid patients attending with uncontrolled ventricular rates. The confounding effect of medication use is likely to be very small, as there were no significant differences in baseline or stress MBF between patients receiving and not receiving β‐blockers or angiotensin‐converting enzyme inhibitors/angiotensin II receptor blockers.

Second, patients with AF had significantly different stress RPP from controls due to limited changes in heart rate and SBP in response to adenosine; this blunted hemodynamic response has been noted previously.^6^ Nevertheless, adenosine stress CMR remains an accurate clinical investigation in patients with AF,^24^ and RPP should not be used to demonstrate adequacy of adenosine stress because the coronary hyperemic effects of adenosine are largely independent of changes in hemodynamics.^16^ Rates of splenic switch‐off, a validated technique for excluding inadequate stress,^14^ were high and similar between groups in our study. Results remained consistent in a sensitivity analysis that excluded subjects without splenic switch‐off.

Third, this study was not designed or powered to investigate the relationship between change in AF burden and change in MBF after ablation. The combination of nonattendance at the follow‐up scan and technical limitations meant that paired pre‐ and postablation comparisons of absolute MBF quantification could be undertaken in only 33 of 49 patients. Most of these patients had a diagnosis of paroxysmal AF and were in SR during both scans; 18 of 33 also experienced at least 1 episode of recurrent AF postablation. Investigation of a larger number of patients with persistent AF and no postablation AF recurrence would have assessed whether baseline MBF improves with recovery of SR postablation, consistent with a direct effect of rhythm on baseline MBF. By contrast, unchanged baseline MBF following successful AF ablation would have been consistent with a primary impairment of MBF, potentially contributing to both myocardial dysfunction and AF. Nevertheless, our study demonstrates that impairment in stress MBF is unrelated to intrascan rhythm, and we achieved adequate power to exclude a clinically relevant improvement in stress MBF after ablation.

Finally, as in previous studies,^6^, ^7^ we did not measure coronary perfusion pressure. Further research is needed to investigate the possibility that lower coronary perfusion pressure relates to reduced myocardial perfusion in patients with AF.

## Conclusions

Baseline and stress myocardial perfusion are significantly impaired in patients with AF but no epicardial coronary artery disease. Lower baseline MBF relates to reduced cardiac performance even after successful ablation, while stress MBF is impaired independently of rhythm during imaging. Coronary microvascular dysfunction may be a relevant pathophysiological mechanism in patients with a history of AF, even after SR has been restored.

## Sources of Funding

This work was supported by the British Heart Foundation through a program grant to Casadei (grant number RG/11/15/29375); the National Institute for Health Research Biomedical Research Centre, Oxford; the European Union's Horizon 2020 Research and Innovation Programme (grant number 633196 [CATCH ME]); the British Heart Foundation Centre of Research Excellence, Oxford (grant number RE/08/004); a British Heart Foundation Clinical Research Training Fellowship (grant number FS/15/11/31233 to Liu).

## Disclosures

Wijesurendra has received a speaker fee/honorarium and travel assistance from Biosense Webster. The remaining authors have no disclosures to report.
